# *Anaplasmataceae-*Specific PCR for Diagnosis and Therapeutic Guidance for Symptomatic Neoehrlichiosis in Immunocompetent Host

**DOI:** 10.3201/eid2202.141762

**Published:** 2016-02

**Authors:** Michael Schwameis, Julia Auer, Dieter Mitteregger, Ingrid Simonitsch-Klupp, Michael Ramharter, Heinz Burgmann, Heimo Lagler

**Affiliations:** Medical University of Vienna, Vienna, Austria

**Keywords:** Candidatus Neoehrlichia, Austria, imported, tick-borne, tickborne, travel-associated, neoehrlichiosis, human, Anaplasmataceae-specific PCR, diagnosis, therapeutic guidance, bacteria, immunocompetent

## Abstract

*Candidatus* Neoehrlichia is increasingly being recognized worldwide as a tickborne pathogen. We report a case of symptomatic neoehrlichiosis in an immunocompetent Austria resident who had recently returned from travel in Tanzania. The use of *Anaplasmataceae*-specific PCR to determine the duration of antimicrobial therapy seems reasonable to avert recrudescence.

Human neoehrlichiosis is an infectious disease that primarily affects immunocompromised persons and persons with severe concurrent medical conditions (*1*–*5*). We describe symptomatic *Candidatus* Neoehrlichia infection in an otherwise healthy woman who had returned from a 28-day vacation in Tanzania, and we illustrate the applicability of *Anaplasmataceae*-specific PCR for diagnosis and therapeutic guidance.

## The Study

In January 2013, a 30-year-old white woman with no relevant medical history was admitted to the Division of Infectious Diseases and Tropical Medicine, General Hospital of Vienna, in Vienna, Austria, because of a 3-week history of high fevers (up to 39.9°C), chills, and night sweats accompanied by headache, muscle pain, and malaise. Four weeks before hospitalization, the woman had returned from a 28-day vacation in Tanzania. She had not taken antimalarial prophylaxis drugs while in Tanzania; instead she carried atovaquone/proguanil tablets as a standby medication. The woman felt well during the entire stay in Tanzania, so she did not take the atovaquone/proguanil. 

Spiking fevers began 5 days after her return to Vienna. She visited the outpatient clinic at the General Hospital of Vienna, where malaria, Dengue virus fever, and typhoid fever were ruled out as causative diseases. During her first days in Tanzania, the woman had voluntary skin contact with a prosimian, but she recalled no recent tick bites or exposures to animals. Blood samples were obtained; multiple cultures were negative. However, over the next 10 days, persistent fever and a deteriorating general condition led to hospitalization for further evaluation. Clinical data at admission and results of primary diagnostic tests are provided in the Table.

**Table:** Clinical data at admission and primary diagnostic test results for a patient with *Candidatus* Neoehrlichia infection, Austria, 2013*

Clinical variable	Finding/value
Subjective symptoms	Malaise, diffuse muscle pain, dull headache (without signs of meningism), and tenderness in the left upper abdominal quadrant
Tympanic temperature	37.8°C, while taking acetaminophen
Heart auscultation	Systolic murmur (right sternal border), tachycardia (125 beats per minute)
Condition of skin	No rash or signs of cutaneous exposure to arthropods
Laboratory testing†	
C-reactive protein	5 mg/dL (<0.5)
Procalcitonin	0.14 ng/mL (<0.5)
Leukocyte count	3.9 × 10^9^/L (4–10)
Neutrophils	53% (50–75)
Lymphocytes	27% (25–40)
Monocytes	16% (0–12)
Fibrinogen	480 mg/dL (180–390)
Serum amyloid A	164 mg/L (<5)
γ-globulins	26.2% (11.1–18.8)
Erythrocyte sedimentation rate	70 mm/h (<15)
Platelet count	121 × 10^9^/L (150–350)
Hemoglobin	9 g/dL (12–16)
Chest radiography	No consolidations, no opacities
Abdominal ultrasonography	Splenomegaly of 15.5 x 6.7 cm
Transesophageal echocardiography	Normal cardiac function and valves, no evidence of vegetations
Cranial computed tomography	Parasagittal meningioma, otherwise normal
Ophthalmologic examination	Bilateral papilloedema
Cerebrospinal fluid	Clear and colorless; absolute cell count 4/μL protein, glucose, and lactate levels within reference range
Abdominal ultrasonography	Splenomegaly, 15.5 × 6.7 cm
Urinary dip stick and urinary cultures	No growth
Blood cultures	No growth
Serologic testing	
HIV	Negative
Hepatitis B and C viruses	Negative
Epstein-Barr virus	Negative
Cytomegalovirus	Negative
*Mycoplasma* spp.	Negative
Adenovirus	Negative
Enterovirus	Negative
Coxsackievirus	Negative
Influenza A,B, and C viruses	Negative
Parainfluenza virus	Negative
*Anaplasma* spp.	Negative
*Rickettsia* spp.	Negative
Tuberculous mycobacteria	Negative
*Plasmodium* spp.	Negative
Syphilis (VDRL, TPPA)	Negative
PCR testing	
*Leishmania* spp.	Negative
*Trypanosoma* spp.	Negative
*Plasmodium* spp.	Negative
Giemsa-stained thin and thick blood smears	
*Plasmodium* spp.	Negative

*TPPA, *Treponema pallidum* particle agglutination assay; VDRL, Venereal Disease Research Laboratory test. †Laboratory data are given as absolute number or percentage (reference range).

During the first days of hospitalization, the woman had fever (up to 39°C) accompanied by general discomfort and headache. Giemsa-stained thick and thin blood smears showed no evidence of malaria. Likewise, serologic and PCR test results for *Plasmodium* spp. were negative.

**Figure 1:**
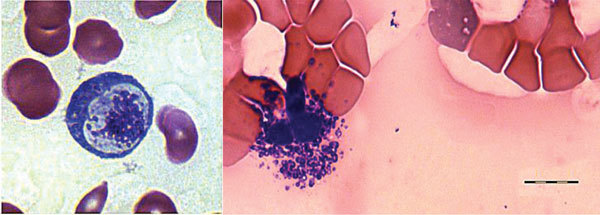
Giemsa-stained blood smear from an immunocompetent patient with *Candidatus* Neoehrlichia infection, Austria, 2013. The blood smear shows possible microbial pathogens within leukocytes. Scale bar indicates 10 μm.

On hospitalization day 5, a peripheral blood sample was tested by using a 16S rRNA gene–based eubacterial broad range PCR (SepsiTest; Molzym GmbH & Co. KG, Bremen, Germany); results were positive. The amplification products (300 bp) were sequenced (GenBank accession no. KT895260) and compared, using BLAST (http://blast.ncbi.nlm.nih.gov/Blast.cgi), with known sequences in the National Center for Biotechnology Information (http://www.ncbi.nlm.nih.gov/) database. The sequence showed 98% (294/300 bp) homology with *Candidatus* Neoehrlichia lotoris (GenBank accession no. EF633744.1; only 1 database entry was available) and 97% (293/301 bp) homology with *Candidatus* Neoehrlichia mikurensis (GenBank accession no. KF155504.1; several database entries were available and showed a reproducible single base deletion at position 225). These findings were confirmed by *Anaplasmataceae*-specific 16S ribosomal RNA gene–based PCR. Primer pairs EHR16SD (5′-GGT ACC YAC AGA AGA AGT CC-3′) and EHR16SR (5′-TAG CAC TCA TCG TTT ACA GC-3′) were chosen to amplify a 345-bp fragment (*6*). The protocol was adjusted to that in the manual for High-Fidelity PCR enzyme mix (Thermo Scientific, Waltham, MA, USA) and to that of Brown et al. (*7*). Bidirectional sequencing of the 345-bp amplicon showed a sequence of 243 bp corresponding to the cDNA strand (GenBank accession no. KT953340) and yielded similar results: 97% (235/243 bp) sequence homology was shared with *Candidatus* Neoehrlichia lotoris (GenBank accession no. EF633744.1), and 96% (235/244 bp) sequence homology was shared with *Candidatus* Neoehrlichia mikurensis (GenBank accession no. JQ359046.1). Because the percentages of shared homologies were not sufficient to attribute the identified microbial agent to an official species, we tentatively named the agent *Candidatus* Neoehrlichia Tanzania. In addition, a microscopy review of Giemsa-stained blood smears obtained within the first days of admission showed structures possibly equivalent to microbial pathogens within leukocytes (Figure 1).

**Figure 2:**
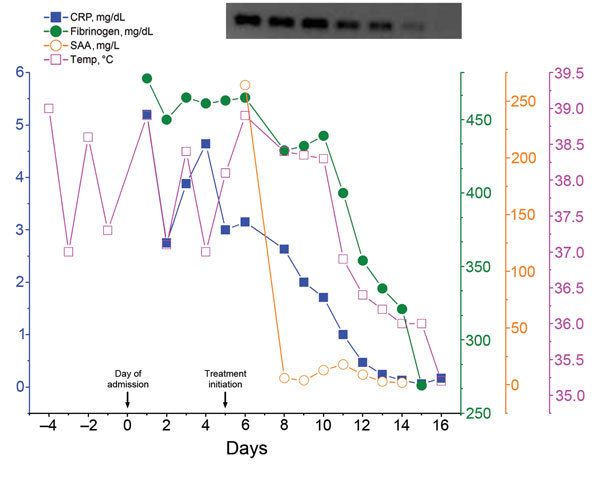
Body temperature and markers of inflammation over the course of hospitalization for a patient with *Candidatus* Neoehrlichia infection, Austria, 2013. Day 0 indicates time of admission. Antimicrobial therapy with doxycycline (300 mg per day) was begun on day 5 and led to a rapid resolution of clinical symptoms and a progressive decrease of all inflammatory parameters. Daily *Anaplasmataceae*-specific PCR measurements guided therapy, which was safely stopped 1 day after disappearance of serum *Candidatus* Neoehrlichia DNA. Upper right shows 1.5% agarose gel electrophoresis analysis. The intensity of the 345-bp DNA band amplified from blood samples progressively decreased over the course of treatment. CRP, C-reactive protein; SAA, serum amyloid A; Temp, tympanic temperature.

Antimicrobial treatment with oral doxycycline (300 mg per day) was subsequently initiated, resulting in improvement in the patient’s overall condition within 2 days and in a continuous decrease of all inflammation markers, normalization of platelet counts, and abatement of fever (Figure 2). However, serum Neoehrlichia DNA remained detectable at high levels. To provide the optimal duration of antibiotic treatment, we performed daily *Anaplasmataceae*-specific 16S PCR measurements of blood samples. Over the next 10 days of therapy, the DNA signal intensity continuously diminished. Doxycycline was stopped 1 day after disappearance of Neoehrlichia serum DNA.

In contrast with patients in previously published reports of human neoehrlichiosis, the patient described in our report was a healthy young woman without concurrent medical conditions. She had signs and symptoms of disease for 4 weeks without any symptomatic improvement before therapy was initiated. Treatment led to a rapid clinical response and rapid clearance of serum Neoehrlichia DNA, which may be attributable to her otherwise good medical condition but may also reflect high antimicrobial efficiency of the high-dose therapeutic regimen applied.

Because symptomatic neoehrlichiosis usually occurs in patients with immunosuppression, we examined the patient for an underlying malignancy or autoimmune disorder. These conditions were largely ruled out by negative test results for HIV and mycobacteria and by a normal finding on 18F-FDG-PET/CT (18F-fluordeoxyglucose-positron emission tomography/computed tomography) examination (except for enhanced splenic FDG uptake). The patient had moderate disease with nonspecific symptoms partly resembling those of human anaplasmosis. The splenomegaly was attributed to polyclonal B cell activity (indicated by hypergammaglobulinemia), but it could also have resulted from direct infection of splenic sinusoidal cells, as found in Neoehrlichia-infected Wistar rats (*8*). However, spleen size decreased over the course of antimicrobial treatment and reached a normal diameter by a 3-week follow-up examination.

No evidence exists regarding the exact incubation period of human neoehrlichiosis, but it probably approximates that of human granulocytic anaplasmosis, suggesting that the patient in our study acquired neoehrlichiosis in Tanzania. Nonetheless, several tickborne diseases are highly endemic in Austria. Glatz et al. (*9*) recently reported a 4.2% prevalence of *Candidatus* Neoehrlichia in *Ixodes ricinus* ticks in Austria. However, in the 5-day period between returning home from Tanzania and fever onset, the patient in our study had stayed in the urban area of Vienna; thus, the possibility that she may have been exposed to ticks in Austria is limited but not excluded. Furthermore, the patient returned to Vienna at the height of winter, making the possible transmission of *Candidatus* Neoehrlichia by a domestic tick even less plausible. On the other hand, no epidemiologic data are available on the prevalence of *Candidatus* Neoehrlichia in ticks in Tanzania, but *Candidatus* Neoehrlichia mikurensis was recently found in ticks of 2 species collected in Nigeria (*10*). Thus, the presence of *Candidatus* Neoehrlichia in ticks in Tanzania and the risk for transmission from ticks to humans seem conceivable. Because a 16S rDNA sequence difference of >2% is arbitrarily considered as indicative for delineation at the species level, it seems possible that a new *Candidatus* Neoehrlichia agent was detected in the patient in our study.

## Conclusions

This case demonstrates that *Candidatus* Neoehrlichia can affect healthy persons who have no underlying hematologic or autoimmune disorders. Neoehrlichiosis should be considered in the differential diagnosis for patients with appropriate symptoms, independent of concurrent conditions and immune status. As long as no evidence-based recommendations regarding treatment of human neoehrlichiosis exist, it seems reasonable to use *Anaplasmataceae*-specific PCR to monitor treatment response and determine the duration of antimicrobial therapy to avert recrudescence.
